# Real-World Treatment Outcomes and Prognostic Factors in Metastatic EGFR-Mutated Non-Small Cell Lung Cancer: A Single-Center Study from a High-Volume Tertiary Chest Hospital

**DOI:** 10.3390/jcm15218206

**Published:** 2026-10-22

**Authors:** Abdülkadir Koçanoğlu, Oktay Ünsal, Fatih Kuş, Nesrin Gürçay, Esra Zeynelgil, Yakup Düzköprü, Serdar Karakaya

**Affiliations:** 1 Medical Oncology, Ankara Atatürk Sanatorium Training and Research Hospital, Ankara 06110, Türkiye; droktayunsal@gmail.com (O.Ü.); fatihkush@hotmail.com (F.K.); drserdarkarakaya@gmail.com (S.K.); 2 Department of Pathology, Ankara Atatürk Sanatorium Training and Research Hospital, Ankara 06110, Türkiye; drgurcaynesrin@gmail.com; 3 Medical Oncology, Ankara Etlik City Hospital, Ankara 06010, Türkiye; esra23.05@hotmail.com; 4 Medical Oncology, Aksaray Training and Research Hospital, Aksaray 68100, Türkiye; yakupduzkopru@gmail.com

**Keywords:** non-small cell lung cancer (NSCLC), epidermal growth factor receptor (EGFR), tyrosine kinase inhibitors, real-world, survival

## Abstract

**Background/Objectives:** Epidermal growth factor receptor (EGFR)-mutated non-small cell lung cancer (NSCLC) is a distinct molecular subgroup responsive to EGFR tyrosine kinase inhibitors (TKIs). This study evaluated real-world treatment outcomes and prognostic factors in patients with EGFR-mutated metastatic NSCLC. **Methods:** This retrospective study included 124 patients treated with first-line EGFR-TKIs between January 2017 and 30 August 2025 at a tertiary center. Progression-free survival (PFS) and overall survival (OS) were estimated using the Kaplan–Meier method, and prognostic factors were assessed using Cox proportional hazards models. **Results:** The median age was 66 years, and 63.7% of patients were female. The most common mutation was exon 19 deletion (65.3%). Median OS for the entire cohort was 31.7 months, and median PFS was 16.3 months. In multivariate analysis, Eastern Cooperative Oncology Group performance status (ECOG PS), histopathological subtype, uncommon EGFR mutations, and bone metastasis were statistically significantly associated with OS in multivariable Cox regression analysis. Among patients receiving second-line treatment, median PFS was 16.7 months with osimertinib and 4.1 months with platinum-based chemotherapy; these nonrandomized treatment-specific outcomes were considered descriptive. **Conclusions:** In conclusion, the survival outcomes observed in our cohort were generally consistent with previously published real-world data in EGFR-mutant metastatic NSCLC. ECOG performance status and bone metastasis were significantly associated with OS, while associations involving histopathological subtype and uncommon EGFR mutations should be considered exploratory given the small subgroup sizes and limited statistical power. These findings provide real-world evidence on treatment patterns and survival outcomes among patients with EGFR-mutant metastatic NSCLC in Türkiye.

## 1. Introduction

Lung cancer continues to be one of the top causes of cancer-related death globally [1]. Roughly 85% of lung cancers are non-small cell lung cancer (NSCLC) [2,3]. Among patients with NSCLC, those harboring epidermal growth factor receptor (EGFR) mutations constitute a distinct molecular subgroup with different therapeutic approaches and prognostic characteristics. The prevalence of EGFR mutations varies geographically, with reported rates of approximately 14.1% in European NSCLC populations and 38.4% in Asian populations [4,5]. Approximately 85–90% of all EGFR mutations consist of exon 19 deletions and exon 21 L858R point mutations. These two alterations are considered the most clinically relevant targetable mutations due to their high sensitivity to EGFR tyrosine kinase inhibitors (TKIs) [6]. Furthermore, it has been demonstrated that rare EGFR mutations such as G719X, S768I, and L861Q react to EGFR TKIs. Exon 20 insertions, on the other hand, are typically regarded as a subgroup resistant to traditional EGFR TKIs [7,8].

EGFR TKIs have become the norm for medical care in EGFR-mutant NSCLC due to their association with prolonged survival, high response rates, and improved quality of life. These agents are classified into three generations based on their development and mechanisms of action. Erlotinib and gefitinib, both first-generation EGFR TKIs, act through reversible inhibition of EGFR kinase activity, whereas second-generation agents, such as afatinib and dacomitinib, form irreversible inhibitory interactions with the receptor [5,9]. As a third-generation EGFR TKI, osimertinib targets common sensitizing EGFR alterations as well as the T790M mutation, a major mechanism of acquired resistance to earlier-generation EGFR inhibitors. It is known that the T790M mutation develops in approximately 50% of patients who have previously received first- or second-generation (1G/2G) EGFR TKIs [10,11].

In the phase III FLAURA trial, first-line osimertinib was evaluated against earlier-generation EGFR TKIs in patients whose tumors harbored an exon 19 deletion or exon 21 L858R substitution. The study reported a median overall survival (OS) of 38.6 months with osimertinib, compared with 31.8 months in the control group, indicating a statistically significant survival benefit (HR 0.80; *p* = 0.046). Nevertheless, subgroup analyses did not demonstrate a statistically significant OS advantage in Asian patients or those harboring the EGFR exon 21 L858R mutation [12]. Real-world data on the treatment of metastatic EGFR-mutant NSCLC show considerable heterogeneity. Studies conducted in Asian populations have generally reported more favorable survival outcomes, whereas some studies from Western populations have demonstrated results inferior to those of the FLAURA trial. Conversely, some real-world analyses have suggested no significant survival difference between first-line osimertinib and sequential use of 1G/2G EGFR TKIs, then osimertinib as the disease progresses [13,14,15,16]. Nevertheless, real-world evidence from different geographic regions consistently supports the efficacy of EGFR TKIs and reinforces their central role in the treatment of EGFR-mutant NSCLC.

In this study, we aimed to evaluate the demographic characteristics, clinical presentation, and real-world treatment outcomes of patients with metastatic EGFR-mutant NSCLC from Türkiye, a country geographically located at the crossroads of Asia and Europe. Additionally, we sought to analyze progression-free survival (PFS) and OS outcomes and identify independent prognostic factors affecting survival and treatment efficacy. We believe that our findings may contribute to the existing literature by providing comparative real-world evidence from different geographic regions.

## 2. Materials and Methods

### 2.1. Data Collection and Patient Follow-Up

Medical records of 139 patients with EGFR-mutated metastatic NSCLC managed at the Medical Oncology outpatient clinics of Ankara Atatürk Sanatorium Training and Research Hospital were retrospectively reviewed. Patients included in the study had initiated first-line EGFR-TKI treatment between 1 January 2017 and 30 August 2025. The final follow-up date was 30 December 2025. Data were retrospectively collected from hospital electronic medical records and patient files. Patients were eligible if they were at least 18 years old, had a histopathologically confirmed diagnosis of NSCLC, received an EGFR TKI as first-line systemic treatment for metastatic disease, and had adequate clinical information and follow-up records available for analysis. Individuals who had already experienced a second primary cancer were excluded. Of the 139 patients initially screened, 15 were excluded: two because of a second primary malignancy, eight because they had received chemotherapy as first-line treatment for metastatic disease, and five because of insufficient clinical data in hospital records. Consequently, 124 patients were included in the final analysis. Among the excluded patients, three had received gefitinib. Two of these patients had received first-line chemotherapy followed by gefitinib in subsequent treatment lines, while the remaining patient was excluded because of insufficient clinical data. Gefitinib use itself was not an exclusion criterion, and the same eligibility criteria were applied to all screened patients.

Information extracted from institutional medical records included demographic characteristics (age, sex, and smoking history), ECOG performance status, histopathological findings, disease stage at diagnosis, EGFR mutation subtype, TP53 and T790M mutation status, PD-L1 expression, comorbidities, and sites of metastasis. Treatment-related data comprised systemic therapies administered, treatment initiation dates, documented progression, last follow-up, and death. EGFR alterations involving G719X in exon 18, L861Q in exon 21, and S768I in exon 20 were categorized as uncommon mutations. Molecular analyses were performed on specimens obtained during the metastatic disease period, before initiation of first-line EGFR-TKI treatment. EGFR mutation status was assessed using next-generation sequencing (NGS) in 21 patients and PCR-based assays in 103 patients. The EGFR PCR assays covered common sensitizing mutations, including exon 19 deletions and exon 21 L858R, as well as uncommon EGFR mutations. TP53 mutation status was available for 42 patients, including 20 assessed by NGS and 22 by PCR-based methods. However, detailed information regarding the specific TP53 regions covered by the PCR assays was not available in the retrospective records. Eight patients harbored uncommon EGFR mutations. No additional EGFR mutations were identified in these patients based on the available molecular assay results; therefore, these alterations were classified as isolated uncommon EGFR mutations. In patients evaluated for acquired T790M resistance after progression on first- or second-generation EGFR-TKIs, T790M testing was performed using either newly obtained tumor tissue or liquid biopsy specimens, depending on specimen availability and clinical feasibility. PD-L1 results were obtained from institutional pathology records.

Radiological tumor assessments were generally performed at approximately 10–14-week intervals as part of routine clinical follow-up. Given the retrospective nature of the study, imaging intervals were not protocol-mandated and could vary according to clinical circumstances. Disease progression was determined based on radiological findings and the documented assessments of the treating clinicians. In cases where disease progression was uncertain, available radiological images were retrospectively reassessed according to RECIST version 1.1. First-line PFS was defined as the interval from EGFR-TKI initiation to the first documented radiological disease progression or death from any cause, whichever occurred first. The date of documented progression was considered the PFS event date, regardless of subsequent treatment continuation or the timing of T790M mutation testing. The overall response rate (ORR) represented the percentage of patients achieving either a complete or partial response, while the disease control rate (DCR) additionally accounted for patients with stable disease. ORR and DCR were calculated using the entire first-line treatment cohort (*n* = 124) as the denominator. All patients underwent at least one radiological response assessment, and no deaths occurred before the first assessment.

For the first-line treatment analysis, PFS was measured from the date of EGFR-TKI initiation until radiologically documented disease progression or death from any cause, whichever occurred first. Patients without documented progression or death were censored at their last radiological disease assessment, which coincided with their final clinical follow-up visit. OS was defined as the time from initiation of first-line EGFR-TKI therapy to death from any cause; patients who were alive at the data cutoff were censored at their last follow-up. Second-line PFS was defined as the time from initiation of second-line systemic therapy to the first documented disease progression or death from any cause, whichever occurred first. Patients without documented progression or death were censored at their last radiological disease assessment, which coincided with their final clinical follow-up visit.

Ethical approval for this retrospective study was obtained from the Ethics Committee of Ankara Atatürk Sanatorium Training and Research Hospital, Ministry of Health, Türkiye (approval no. 2024-BÇEK/377; 24 September 2025). The research was carried out in accordance with the ethical principles outlined in the Declaration of Helsinki.

### 2.2. Statistical Analysis

All analyses were conducted with IBM SPSS Statistics, version 22.0 (IBM Corp., Armonk, NY, USA). Categorical data are presented as frequencies and percentages, while continuous measurements are reported as means with standard deviations or medians with minimum and maximum values, depending on the distribution of the data.

Kaplan–Meier estimates were used to characterize PFS and OS, and differences between survival curves were assessed with the log-rank test. The *p*-values displayed in the Kaplan–Meier survival figures were derived from log-rank tests and represent unadjusted comparisons between groups. Adjusted hazard ratios and corresponding *p*-values obtained from multivariable Cox proportional hazards regression analyses are presented separately in tables. Follow-up duration was calculated using the reverse Kaplan–Meier approach. Associations between potential prognostic variables and survival endpoints were initially examined through univariable Cox proportional hazards regression.

Variables associated with survival outcomes at *p* < 0.05 in univariable Cox regression analyses were included in the corresponding primary multivariable models, provided that sufficient data were available. PD-L1 expression and TP53 mutation status were excluded from these models because of incomplete data availability. T790M mutation status was also excluded because of incomplete data and its assessment after disease progression, which precluded its use as a baseline prognostic variable. No missing data were present for the covariates included in the primary multivariable models, and no imputation was performed. To further address potential confounding and assess the robustness of the findings, supplementary multivariable Cox regression analyses were performed using clinically selected covariates irrespective of their statistical significance in univariable analyses. These covariates included sex, smoking status, ECOG performance status, histopathological group, EGFR mutation subtype, brain, liver, bone, and pleural metastases, and metastatic disease at initial diagnosis. The covariates were selected to account for potentially relevant demographic, clinical, histopathological, molecular, and disease-related prognostic factors. All covariates were entered simultaneously into the supplementary models.

Hazard ratios (HRs) were presented with 95% confidence intervals (CIs). The proportional hazards assumption was evaluated using scaled Schoenfeld residuals for individual covariates and globally. Variance inflation factors (VIFs) were used to evaluate multicollinearity among the variables in the multivariable models. Potential model overfitting was evaluated by considering the number of outcome events relative to the number of regression parameters. Given the small sizes of selected subgroups, sensitivity analyses were performed by excluding, where appropriate, patients treated with dacomitinib (*n* = 3), patients with non-adenocarcinoma histology (*n* = 6), and patients with uncommon EGFR mutations (*n* = 8) to evaluate the robustness of the multivariable estimates. All statistical tests were two-sided, and a *p*-value < 0.05 was considered statistically significant.

## 3. Results

A total of 124 patients were included in the study, of whom 79 (63.7%) were female. The median age at diagnosis was 66 years (range: 30–86). Sixty-nine patients (55.7%) had at least one comorbid condition. Mucin staining was positive in 55 patients (44.4%). The most common mutation was exon 19 deletion, identified in 81 patients (65.3%). A total of 88 patients (71%) presented with de novo metastatic disease. The most frequent metastatic site was the pleura, observed in 74 patients (59.7%), followed by bone metastases in 62 patients (50%). Brain metastases were present in 29 patients (23.4%). Liver, distant lymph node, and contralateral lung metastases were observed in 16 (12.9%), 29 (23.4%), and 47 (37.9%) patients, respectively (Table 1).

Among the 124 patients, 36 (29.0%) were initially diagnosed with non-metastatic NSCLC and subsequently developed metastatic disease. Of these, 24 patients (66.7%) underwent surgical resection, while 12 patients (33.3%) received definitive chemoradiotherapy. Among the surgically treated patients, 18 (75.0%) also received adjuvant chemotherapy. The median interval from initial diagnosis to the development of metastatic disease, estimated using the Kaplan–Meier method, was 23.45 months (95% CI: 17.08–29.83).

Erlotinib was the most commonly used first-line therapy, administered to 71 patients (57.3%). Afatinib, osimertinib, and dacomitinib were used in 36 (29%), 14 (11.3%), and 3 (2.4%) patients, respectively. Among the eight patients with uncommon EGFR mutations, seven received first-line afatinib, while one patient harboring an exon 18 G719X mutation received dacomitinib. Patients with EGFR exon 20 insertions were not represented in our cohort, as they did not receive first-line EGFR-TKI therapy during the study period. Baseline demographic, clinical, histopathological, and molecular characteristics, together with treatment initiation periods, are presented according to first-line EGFR-TKI in Supplementary Table S1.

The median follow-up duration, calculated using the reverse Kaplan–Meier method, was 44.8 months (95% CI: 25.98–63.64) in the overall cohort. According to first-line EGFR-TKI treatment, the median follow-up durations were 53.1 months (95% CI: 25.79–80.45) for erlotinib, 54.8 months (95% CI: 42.41–67.19) for afatinib, 11.1 months (95% CI: 10.16–12.17) for osimertinib, and 18.1 months (95% CI: not estimable) for dacomitinib.

**Figure 1:**
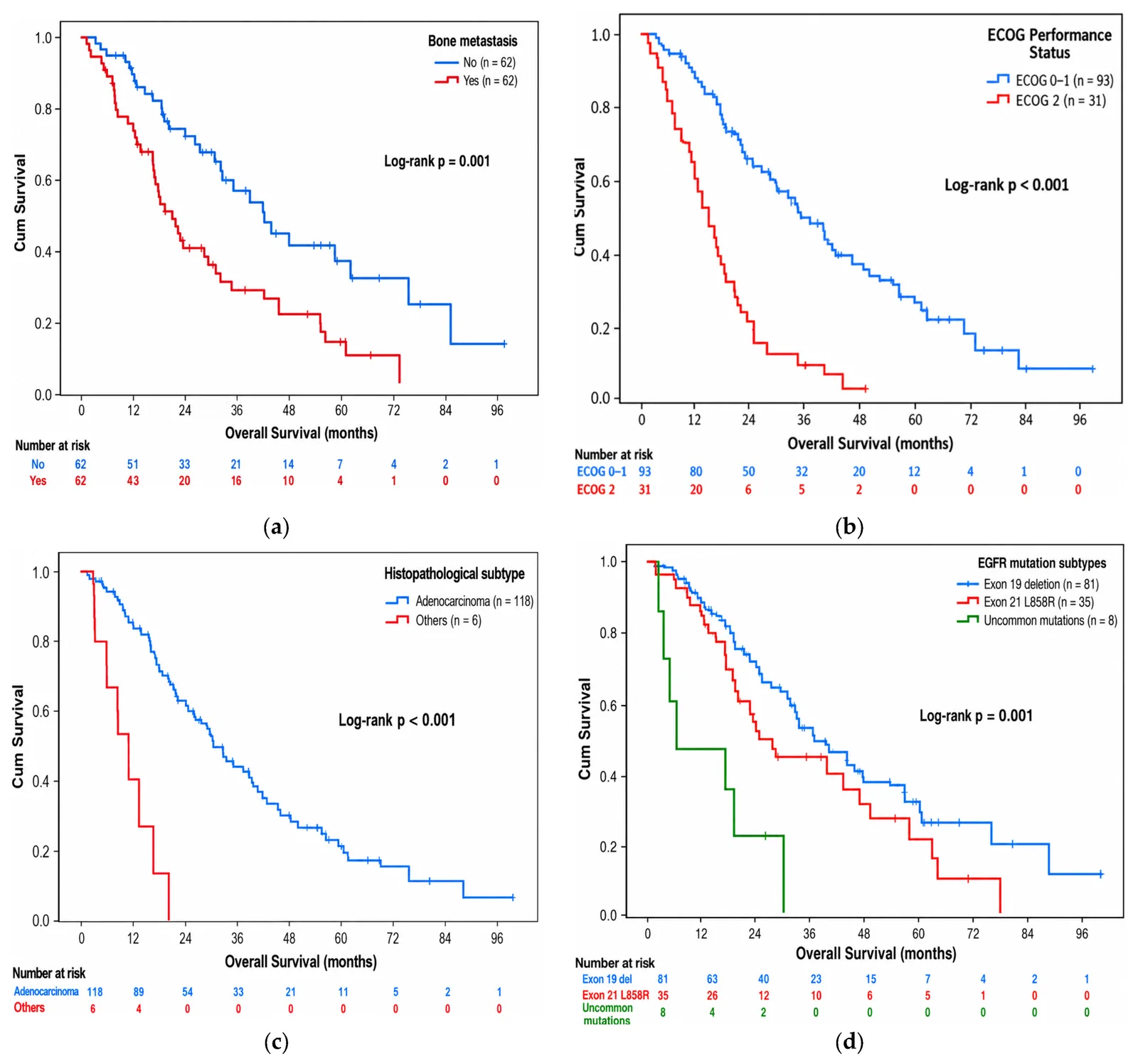
Kaplan–Meier curves for overall survival according to prognostic factors: (**a**) bone metastasis; (**b**) ECOG performance status; (**c**) histopathological subtype; and (**d**) EGFR mutation subtype.

In the entire cohort, median OS was 31.7 months (95% CI: 23.4–43.5). Median OS was not reached in the osimertinib and dacomitinib groups, whereas it was 34.4 months (95% CI: 25.43–55.80) in the erlotinib group and 26.5 months (95% CI: 19.42–45.3) in the afatinib group. The difference between treatment groups was not statistically significant (*p* = 0.545). In multivariable analysis, ECOG performance status, histopathological subtype, EGFR mutation subtype, and presence of bone metastasis were independently associated with OS (Table 2). Kaplan–Meier survival curves for statistically significant variables are shown in Figure 1.

**Figure 2:**
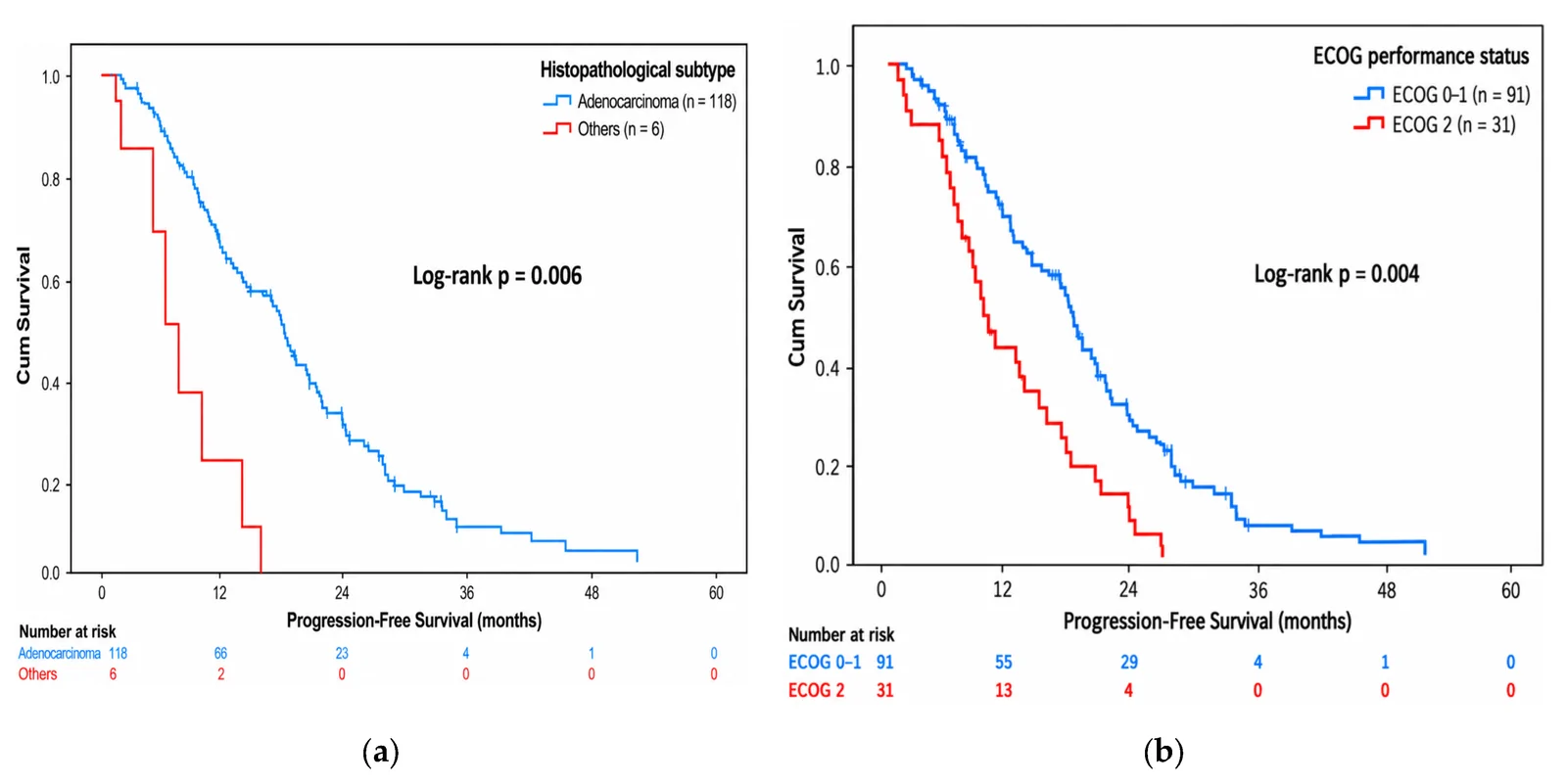
Kaplan–Meier curves for progression-free survival according to prognostic factors: (**a**) ECOG performance status; (**b**) histopathological subtype.

The median PFS for the entire cohort was 16.3 months (95% CI: 12.7–18.2). Median PFS was not reached in the osimertinib group, whereas it was 16.3 months (95% CI: 13.01–19.60), 15.1 months (95% CI: 11.04–22.40), and 17.8 months (95% CI: 4.21–not available) in the erlotinib, afatinib, and dacomitinib groups, respectively. No statistically significant difference was observed among treatment groups (*p* = 0.963). At 12 months, PFS rates were 59% for erlotinib, 55% for afatinib, 66% for dacomitinib, and 71% for osimertinib. In multivariable analysis, ECOG performance status and histopathological subtype remained significantly associated with PFS, whereas pleural metastasis was not independently associated with PFS (Table 3). Kaplan–Meier progression-free survival curves for statistically significant variables are shown in Figure 2.

In supplementary multivariable Cox regression analyses incorporating clinically selected covariates, ECOG performance status and histopathological group remained significantly associated with both OS and PFS. Bone metastasis and EGFR mutation subtype were significantly associated with OS, whereas pleural metastasis remained significantly associated with PFS (Supplementary Table S2). Given the small sample sizes and heterogeneity of the “Others” histopathological group and the uncommon EGFR mutation subgroup, the corresponding associations should be interpreted as exploratory.

During follow-up, 74 deaths (59.7%) were recorded in the overall cohort. According to first-line EGFR-TKI treatment, deaths occurred in 44 of 71 patients (62.0%) receiving erlotinib, 27 of 36 (75.0%) receiving afatinib, 2 of 14 (14.3%) receiving osimertinib, and 1 of 3 (33.3%) receiving dacomitinib. No significant violation of the proportional hazards assumption was identified for individual covariates or globally in the multivariable OS model (global Schoenfeld test, *p* = 0.639). No relevant multicollinearity was detected, with all VIF values < 1.1. With 74 events and five regression parameters, the event-per-parameter ratio was 14.8.

A total of 106 PFS events (85.5%) were recorded during follow-up. By first-line EGFR-TKI treatment, PFS events occurred in 65 of 71 patients (91.5%) receiving erlotinib, 35 of 36 (97.2%) receiving afatinib, 4 of 14 (28.6%) receiving osimertinib, and 2 of 3 (66.7%) receiving dacomitinib. No significant violation of the proportional hazards assumption was identified for individual covariates or globally in the multivariable PFS model (global Schoenfeld test, *p* = 0.343). All VIF values were <1.1. With 106 events and three regression parameters, the event-per-parameter ratio was 35.3.

Sensitivity analyses were performed to evaluate the potential influence of small subgroups. After exclusion of the three dacomitinib-treated patients, the associations in the OS model remained consistent for ECOG PS 2 (HR 3.95, *p* < 0.001), non-adenocarcinoma histology (HR 4.18, *p* = 0.004), uncommon EGFR mutations (HR 3.35, *p* = 0.009), and bone metastasis (HR 2.21, *p* = 0.003). Exclusion of patients with non-adenocarcinoma histology or uncommon EGFR mutations similarly did not materially alter the associations of ECOG performance status and bone metastasis with OS. For PFS, the association with ECOG performance status remained consistent across sensitivity analyses. After exclusion of dacomitinib-treated patients, the effect estimate for non-adenocarcinoma histology remained directionally similar (HR 2.36), although statistical significance was attenuated (*p* = 0.069), indicating reduced precision associated with the small size of this subgroup.

Following first-line EGFR-TKI treatment, 59 patients (47.6%) received second-line systemic therapy, including 38 treated with osimertinib, 20 with platinum-based doublet chemotherapy, and one with amivantamab plus lazertinib within a clinical trial. Among patients receiving second-line osimertinib, 24 had initially received erlotinib and 14 had received afatinib, while none had received dacomitinib as first-line treatment. Third-line systemic therapy was administered to 17 patients (13.7%), including three treated with osimertinib, 13 with platinum-based doublet chemotherapy, and one with amivantamab plus lazertinib within a clinical trial. Of the three patients receiving third-line osimertinib, two had initially received erlotinib and one had received afatinib; all three had received platinum-based doublet chemotherapy as second-line treatment. No patients received immunotherapy. Palliative radiotherapy was administered to 18 patients following first-line progression, six following second-line progression, and four following third-line progression.

We have also clarified the relationship between T790M mutation status and subsequent-line osimertinib use. Among the 41 patients who received osimertinib in the second or third line, 38 were T790M-positive (36 in the second line and two in the third line), while three were T790M-negative (two in the second line and one in the third line). Thus, all 38 T790M-positive patients received subsequent-line osimertinib, and the difference between the number of T790M-positive patients and subsequent-line osimertinib recipients is explained by the three additional T790M-negative patients.

Among patients receiving second-line treatment, median second-line PFS was 16.7 months (95% CI: 9.24–24.20) in 38 patients treated with osimertinib compared with 4.1 months (95% CI: 0.51–9.58) in 20 patients treated with platinum-based chemotherapy (*p* < 0.001). However, treatment allocation was not randomized and was influenced by T790M mutation status, resulting in potential selection bias between the treatment groups. Therefore, the observed differences in PFS should be regarded as descriptive numerical findings rather than evidence of comparative treatment efficacy.

T790M mutation status was available for 75 patients (60.5%), of whom 38 (30.6% of the total cohort) were T790M-positive. Among patients tested for T790M, the positivity rate was 50.4%.

Among 42 patients with known p53 status, median PFS was 12.6 months (95% CI: 9.01–16.22) in p53-mutant patients and 19.8 months (95% CI: 15.76–23.93) in p53 wild-type patients, with no statistically significant difference (*p* = 0.293). Regarding OS, median survival was 17.8 months (95% CI: 8.08–27.60) in the p53-mutant group, while median OS was not reached in the p53 wild-type group (*p* = 0.068).

All 124 patients underwent at least one radiological response assessment. In the overall cohort, ORR was 82.2% (102/124), and DCR was 91.1% (113/124). No patients died before their first radiological assessment. ORRs and DCRs according to first-line EGFR-TKI treatment are summarized in Table 4.

## 4. Discussion

Our analysis examined survival outcomes and their associated prognostic factors in a real-world cohort of patients with EGFR-mutated metastatic NSCLC who initiated treatment with an EGFR TKI. ECOG PS, histopathological subtype, uncommon EGFR mutations, and the presence of bone metastasis were identified as significant prognostic factors for OS. In our cohort, median OS for the overall population was 31.7 months. Median OS was 34.4 months in patients treated with erlotinib and 26.5 months in those treated with afatinib, whereas median OS was not reached in patients receiving osimertinib or dacomitinib. In the FLAURA trial, median OS was 38.6 months in the osimertinib arm and 31.8 months in the comparator arm receiving 1G/2G EGFR TKIs. Notably, no OS difference was observed among Asian patients and those with exon 21 L858R mutations [12]. These findings established osimertinib as the new standard first-line therapy. However, the limited OS benefit observed in certain subgroups and regional differences in drug accessibility have led to increasing interest in sequential treatment strategies involving 1G/2G EGFR TKIs followed by osimertinib upon progression in real-world settings.

Uehara et al. reported a median OS of 33.7 months in the osimertinib group and 41.8 months in patients initially treated with 1G/2G EGFR TKIs in a Japanese cohort, with no statistically significant difference between the groups (*p* = 0.62) [17]. In a Taiwanese real-world study, patients receiving osimertinib after afatinib had a median OS of 55 months, compared with 32.3 months in those receiving chemotherapy after afatinib; the overall median OS for the cohort was 42 months [18]. Kim et al. reported a median OS of 35.6 months in patients treated with 1G/2G EGFR TKIs and 47.6 months in those receiving osimertinib first-line, demonstrating a significant survival benefit in favor of osimertinib (*p* = 0.015) [19]. In a U.S. cohort, Sabari et al. reported a median OS of 28.6 months in patients receiving first-line osimertinib, whereas Nieva et al. reported a median OS of 26.4 months in patients treated with 1G/2G EGFR TKIs. 32% of patients in the Nieva et al. research had brain metastases at the time of diagnosis. Notably, 36.1% of patients in the population Sabari et al. investigated had brain metastases, 15.1% had liver metastases, and 17.1% had ECOG PS ≥ 2 [20,21]. In a UK-based real-world study by Gomez-Randulfe et al., median OS was 16.59 months in the osimertinib group and 16.92 months in the 1G/2G EGFR-TKIs group, with no statistically significant difference (*p* = 0.96). The poor survival outcomes in both groups were attributed to a high proportion of patients with ECOG PS ≥ 2 (32.7%) and a relatively high rate of uncommon EGFR mutations (14.3%) [15]. Overall, our survival outcomes are broadly consistent with previously published real-world data and the results reported in the FLAURA trial. In Western real-world studies, median OS has generally been lower than that reported in the FLAURA trial, whereas Asian cohorts have often demonstrated more favorable outcomes. Our results appear to fall between these two ranges. In our cohort, most patients received first-line 1G/2G EGFR TKIs. However, the small number of patients who received first-line osimertinib and the relatively short median follow-up in this subgroup preclude a meaningful comparative assessment of survival outcomes between first-line osimertinib and earlier-generation EGFR TKIs.

An important consideration when interpreting real-world survival outcomes is the difference in baseline characteristics between clinical trial populations and unselected patients treated in routine clinical practice. In the FLAURA trial, patients were required to have a WHO performance status of 0–1, whereas our cohort included 32 patients (25.8%) with ECOG PS 2. Furthermore, brain, bone, and liver metastases were present in 23.4%, 50.0%, and 12.9% of our patients, respectively. In our study, ECOG PS 2 and bone metastases were independently associated with poorer overall survival [12]. These findings suggest that differences in baseline performance status and metastatic disease burden may partly explain the variability in survival outcomes across real-world cohorts, highlighting the importance of patient characteristics when interpreting treatment outcomes in routine clinical practice.

The therapeutic landscape of EGFR-mutated NSCLC has evolved substantially during the study period. The FLAURA 2 trial demonstrated that first-line osimertinib plus platinum–pemetrexed significantly improved overall survival compared with osimertinib monotherapy (median OS, 47.5 vs. 37.6 months; HR, 0.77; *p* = 0.02) [22]. Similarly, the final overall survival analysis of the MARIPOSA trial demonstrated a significant survival benefit with first-line amivantamab plus lazertinib compared with osimertinib (HR for death, 0.75; 3-year OS, 60% vs. 51%) [23]. Emerging approaches, including CD24-directed strategies such as ATG-031 in combination with EGFR TKIs, are also under investigation [24]. These developments highlight the rapidly evolving treatment landscape and provide important context for interpreting our real-world findings, which largely reflect an earlier era of EGFR-TKI treatment.

Although median OS had not yet been reached in the osimertinib group, 12-month PFS rates were numerically higher in this group (71%) than in patients treated with first- or second-generation EGFR TKIs (55–59%). Nevertheless, the small sample size and shorter follow-up duration of the osimertinib subgroup preclude meaningful comparisons between treatment strategies. Zhang et al. reported a median PFS of 19.4 months with osimertinib and 10.9 months with 1G/2G EGFR TKIs [13]. Another study from East Asia reported median PFS values of 18.8 months and 13.1 months for osimertinib and afatinib, respectively [25]. In our study, the PFS outcomes observed with afatinib and erlotinib were somewhat longer than those reported in several previous studies. Differences in baseline patient characteristics and the retrospective nature of disease progression assessment may have contributed to these variations. However, these potential explanations remain speculative.

In our cohort, median second-line PFS was 16.7 months among patients receiving osimertinib and 4.1 months among those receiving platinum-based chemotherapy. In the AURA2 and AURA3 trials, median PFS with osimertinib was 9.9 and 10.1 months, respectively, while median PFS in the chemotherapy arm of AURA3 was 4.4 months [26,27]. Real-world studies from Italy and Taiwan reported median second-line PFS durations of 9.8 and 11 months with osimertinib, respectively [18,28]. Although the median PFS observed with second-line osimertinib in our cohort was numerically longer than that reported in these studies, differences in patient selection, clinical characteristics, and treatment pathways limit cross-study comparisons. Furthermore, the osimertinib and chemotherapy groups in our cohort were not directly comparable because treatment selection was influenced by T790M mutation status and other clinical factors. Accordingly, these findings should be interpreted as descriptive treatment-specific outcomes rather than evidence of comparative efficacy.

In both univariable and multivariable analyses, ECOG performance status and histopathological subtype emerged as significant prognostic factors for PFS. For OS, ECOG performance status, histopathological subtype, uncommon EGFR mutations, and bone metastasis were identified as independent prognostic factors. Terashima et al. similarly reported bone metastasis as a negative prognostic factor, suggesting that increased tumor burden may explain this association [29]. Although brain metastasis has been identified as a prognostic factor in several studies, we observed only a numerical trend without statistical significance [28,30]. Liang et al. reported better survival in patients with adenocarcinoma histology and exon 19 deletions compared with other subtypes and exon 21 L858R mutations [31]. Although previous studies have reported more favorable outcomes in patients with EGFR exon 19 deletions and poorer outcomes in those harboring uncommon EGFR mutations, our study identified a significant association with shorter OS only in the uncommon EGFR mutation subgroup [7,20,32,33]. However, the associations observed for histopathological subtype and uncommon EGFR mutations warrant cautious interpretation. The “Others” histopathological category comprised only six patients with heterogeneous diagnoses, including NSCLC-NOS, which does not represent a definitive non-adenocarcinoma subtype. Similarly, the uncommon EGFR mutation subgroup included only eight patients, six of whom harbored S768I mutations, limiting the generalizability of the findings. The limited representation of these subgroups is also evident in pivotal trials. In FLAURA, only nine patients were classified as having “Other” histology, while approximately 97% of patients in the principal treatment arms of MARIPOSA had adenocarcinoma histology. Furthermore, these trials primarily enrolled patients with EGFR exon 19 deletions or L858R mutations, limiting the available evidence regarding uncommon EGFR mutations [12,23]. Therefore, the associations observed in these small subgroups should be regarded as exploratory and hypothesis-generating rather than definitive evidence of independent prognostic effects.

In a subgroup analysis of patients with known p53 mutation status, median OS was 17.8 months in p53-mutant patients, whereas median OS was not reached in wild-type patients, although the difference was not statistically significant. Previous studies have suggested that p53 mutations are associated with poor prognosis in EGFR-mutant NSCLC [20,34,35]. The lack of statistical significance in our study may be due to the limited number of patients with available p53 data.

Several methodological limitations should be considered when interpreting our findings. The retrospective analysis of patients from a single institution involved variations in clinical characteristics, imaging schedules, molecular testing practices, and subsequent treatment pathways. The first-line osimertinib subgroup comprised relatively few patients, and follow-up in this subgroup was substantially shorter than that of the overall cohort, limiting reliable comparisons of long-term survival across first-line EGFR-TKI strategies. Some clinicopathological subgroups, particularly non-adenocarcinoma histology and uncommon EGFR mutations, also included relatively few patients. Although the overall number of events relative to model complexity was adequate and the principal OS associations remained consistent in sensitivity analyses, HR estimates for these small subgroups should be interpreted cautiously because of limited precision. This was particularly relevant to the association between non-adenocarcinoma histology and PFS. Biomarker data, particularly TP53 and PD-L1, were unavailable for a substantial proportion of patients, and analyses involving these variables should therefore be considered exploratory. Tumor response assessments were retrospectively derived from routine clinical records rather than from a prospective protocol or blinded central radiological review. Detailed clinical characteristics at second-line treatment initiation were not consistently available in the retrospective records, limiting our ability to assess differences in prognostic factors between the second-line treatment groups. Furthermore, treatment allocation was influenced by T790M mutation status and was not randomized. Therefore, the second-line treatment outcomes should be interpreted descriptively and should not be considered evidence of comparative efficacy. Finally, exclusion of the small gefitinib-treated subgroup despite inclusion of other small treatment groups may have introduced additional selection bias. Furthermore, as our retrospective study included only EGFR-mutated patients receiving first-line EGFR-TKIs, the prevalence of EGFR mutations among all molecularly tested NSCLC patients at our institution could not be determined. Larger multicenter studies with longer and more balanced follow-up are needed to validate these findings. Nevertheless, the single-center nature of our study may have minimized variations in clinical practice and reduced the likelihood of missing data, thereby improving the consistency of data collection and patient management.

## 5. Conclusions

In conclusion, the survival outcomes observed in our cohort were generally consistent with previously published real-world data in EGFR-mutant metastatic NSCLC. ECOG performance status and bone metastasis were significantly associated with OS, while associations involving histopathological subtype and uncommon EGFR mutations should be considered exploratory given the small subgroup sizes and limited statistical power. These findings provide real-world evidence on treatment patterns and survival outcomes among patients with EGFR-mutant metastatic NSCLC in Türkiye. However, the limited number of patients receiving first-line osimertinib and the shorter follow-up in this subgroup preclude definitive comparisons between first-line treatment strategies.

## Figures and Tables

**Table 1:** Clinicopathological characteristics of 124 patients.

Features	Frequency n (%)
Gender	
Female	79 (63.7)
Male	45 (36.3)
ECOG ^1^ Performance Status	
0	4 (3.2)
1	88 (70.9)
2	32 (25.9)
Smoking status	
No	91 (73.3)
Yes	33 (26.6)
Histopathological subtype	
Adenocarcinoma	118 (95.2)
Squamous cell carcinoma	2 (1.6)
NOS ^2^	3 (2.4)
Adenosquamous carcinoma	1 (0.8)
Adenocarcinoma subtypes	
Acinar predominant	23(18.5)
Papillary predominant	6(4.8)
Lepidic predominant	4(3.2)
Micropapillary predominant	1(0.8)
Solid predominant	2(1.6)
Unknown	82(66.1)
EGFR mutation ^3^	
Exon 19 deletion	81 (65.3)
Exon 21 L858R	35 (28.2)
Exon 18 G719X	1 (0.8)
Exon 21 L861Q	1 (0.8)
Exon 20 S768I	6 (4.8)
First-line treatment	
Erlotinib	71 (57.3)
Afatinib	36 (29)
Osimertinib	14 (11.3)
Dacomitinib	3 (2.4)
De novo metastasis	
No	36 (29.0)
Yes	88 (71.0)
Site of metastasis	
Contralateral Lung	47 (37.9)
Bone	62 (50)
Liver	16 (12.9)
Brain	29 (23.4)
PD-L1 ^4^ groups	
PD-L1 < 1%	28 (22.6)
PD-L1 1–49%	12 (9.7)
PD-L1 ≥ 50%	5 (4)
Unknown	79 (63.7)
P53 status	
Mutant	15 (12.1)
Wild	27 (21.8)
Not tested	82 (66.1)
T790M status	
Mutant	38 (30.6)
Wild	37 (29.9)
Not tested	49 (39.5)

^1^ Eastern Cooperative Oncology Group, ^2^ Not Otherwise Specified, ^3^ All uncommon EGFR mutations were detected as isolated alterations, without concurrent EGFR mutations, ^4^ Programmed Death-Ligand 1.

**Table 2:** Prognostic factors on overall survival with univariable and multivariable analysis.

Features		Median (Months)	Univariable HR (95% CI), *p* Value	Multivariable HR (95% CI), *p* Value
Gender	Male	34.4	1.01 (0.61–1.67) *p* = 0.955	
	Female	31.3		
Smoking history	Yes	29.1	1.08 (0.64–1.80) *p* = 0.779	
	No	31.7		
ECOG PS	0–1	45.3	4.01 (2.38–6.78) *p* < 0.001 *	3.91 (2.29–6.66) *p* < 0.001 *
	2	17.5		
Histopathological subtype	Adenocarcinoma	34.3	4.95 (2.05–11.96) *p* < 0.001 *	5.11 (2.09–12.52) *p* < 0.001 *
	Others	13.9		
Mucin staining	Present	41.6	1.45 (0.91–2.32) *p* = 0.116	
	Absent	25.4		
EGFR subtypes	Exon 19 del	34.3	1.35 (0.81–2.25) *p* = 0.243	1.50 (0.89–2.55) *p* = 0.129
	Exon 21 L858R	26.5		
	Uncommon mutations	11.2	4.41 (1.94–10.04) *p* < 0.001 *	4.03 (1.73–9.40) *p* = 0.001 *
De novo metastatic	Yes	31.7	1.17 (0.73–1.88) *p* = 0.521	
	No	31.7		
Brain metastasis	Yes	20.6	1.62 (0.95–2.78) *p* = 0.079	
	No	34.4		
Liver metastasis	Yes	28.2	1.46 (0.77–2.78) *p* = 0.249	
	No	31.7		
Distant lymph node metastasis	Yes	30.8	1.26 (0.73–2.19) *p* = 0.402	
	No	31.7		
Bone metastasis	Yes	21.4	2.20 (1.36–3.54) *p* = 0.001 *	2.05 (1.24–3.42) *p* = 0.006 *
	No	41.9		
Pleural metastasis	Yes	29.8	0.75(0.47–1.20) *p* = 0.237	
	No	34.3		
Contralateral lung metastasis	Yes	28.2	1.53 (0.96–2.44) *p* = 0.076	
	No	41.9		

* Represents a significant outcome (*p* < 0.05). HRs > 1 indicate an increased hazard relative to the reference category. Reference categories were ECOG PS 0–1, adenocarcinoma histology, exon 19 deletion for EGFR mutation subtype, absence of the respective metastatic site, and the corresponding “No” category where applicable. ECOG PS, Eastern Cooperative Oncology Group Performance Status; HR, hazard ratio; CI, confidence interval; EGFR, Epidermal Growth Factor Receptor.

**Table 3:** Prognostic factors on progression-free survival with univariable and multivariable analysis.

Features		Median (Months)	Univariable HR (95% CI), *p* Value	Multivariable HR (95% CI), *p* Value
Gender	Male	16.0	1.10 (0.73–1.66) *p* = 0.637	
	Female	17.1		
Smoking history	Yes	16.5	0.83 (0.54–1.28) *p* = 0.398	
	No	16.1		
ECOG PS	0–1	17.3	1.90 (1.22–2.96) *p* = 0.004 *	1.75 (1.12–2.75) *p* = 0.014 *
	2	10.1		
Histopathological subtype	Adenocarcinoma	16.9	3.08 (1.32–7.21) *p* = 0.010 *	2.79 (1.19–6.53) *p* = 0.018 *
	Others	8.8		
Mucin staining	Present	17.3	1.17 (0.79–1.73) *p* = 0.429	
	Absent	13.8		
EGFR subtypes	Exon 19 del	17.1	1.19 (0.77–1.84) *p* = 0.439 1.75 (0.80–3.82) *p* = 0.162	
	Exon 21 L858R	16.5		
	Uncommon mutations	6.5		
First-line treatment	Erlotinib	16.3	1.11 (0.73–1.69) *p* = 0.614 1.13 (0.40–3.21) *p* = 0.812 1.09 (0.26–4.49) *p* = 0.906	
	Afatinib	15.1		
	Osimertinib	NR		
	Dacomitinib	17.8		
De novo metastatic	Yes	17.1	0.81 (0.52–1.26) *p* = 0.343	
	No	13.7		
Brain metastasis	Yes	13.3	1 (0.63–1.58) *p* = 0.998	
	No	16.5		
Liver metastasis	Yes	10.5	1.14 (0.66–1.98) *p* = 0.639	
	No	16.5		
Distant lymph node metastasis	Yes	10.8	1.26 (0.79–1.99) *p* = 0.328	
	No	17.1		
Bone metastasis	Yes	11.2	1.28 (0.87–1.88) *p* = 0.215	
	No	18.1		
Pleural metastasis	Yes	13.8	1.55 (1.04–2.32) *p* = 0.033 *	1.43 (0.95–2.15) *p* = 0.089
	No	17.7		
Contralateral lung metastasis	Yes	15.5	0.93 (0.62–1.39) *p* = 0.720	
	No	16.6		

* Represents a significant outcome (*p* < 0.05). HRs > 1 indicate an increased hazard relative to the reference category. Reference categories were ECOG PS 0–1, adenocarcinoma histology, exon 19 deletion for EGFR mutation subtype, erlotinib, absence of the respective metastatic site, and the corresponding “No” category where applicable. ECOG PS, Eastern Cooperative Oncology Group Performance Status; HR, hazard ratio; CI, confidence interval; EGFR, Epidermal Growth Factor Receptor; NR, not reached.

**Table 4:** ORR and DCR results.

n (%)	Erlotinib	Afatinib	Osimertinib	Dacomitinib	Total	*p* Value
	71 (57.3)	36 (29.0)	14 (11.3)	3 (2.4)	124 (100)	
ORR	60 (84.5)	27 (75)	13 (92.9)	2 (66.7)	102 (82.2)	0.373
DCR	67 (94.4)	31 (86.1)	13 (92.9)	2 (66.7)	113 (91.1)	0.229
	71 (57.3)	36 (29.0)	14 (11.3)	3 (2.4)	124 (100)	

ORR, Objective Response Rate; DCR, Disease Control Rate.

## Data Availability

The data generated and/or analyzed in this study are included within this article.

## Supplementary Materials

The following supporting information can be downloaded at: https://www.mdpi.com/article/10.3390/jcm15218206/s1, Supplementary Table S1. Baseline characteristics and treatment initiation periods according to first-line EGFR-TKI; Supplementary Table S2. Multivariable Cox regression analyses of overall survival and progression-free survival using clinically selected covariates.

## Abbreviations

The following abbreviations are used in this manuscript:

NSCLC	Non-Small Cell Lung Cancer
EGFR	Epidermal Growth Factor Receptor
TKI	Tyrosine Kinase Inhibitor
1G/2G	First or Second Generation
HR	Hazard Ratio
PFS	Progression-Free Survival
OS	Overall Survival
ECOG PS	Eastern Cooperative Oncology Group Performance Status
PD-L1	Programmed Death-Ligand 1
SPSS	Statistical Package for the Social Sciences
NOS	Not Otherwise Specified
NR	Not Reached
CI	Confidence Interval
ORR	Objective Response Rate
DCR	Disease Control Rate
