# Supplementary material for: Real-World Treatment Outcomes and Prognostic Factors in Metastatic EGFR-Mutated Non-Small Cell Lung Cancer: A Single-Center Study from a High-Volume Tertiary Chest Hospital
Source: J Clin Med. 2026 Oct 22;15(21):8206. doi: 10.3390/jcm15218206 (PMC13764168; doi:10.3390/jcm15218206)
Supplement: Supplementary file 1 [file jcm-15-08206-s001.zip › jcm-4575864-supplementary.pdf]

Supplementary Table S1. Baseline characteristics and treatment initiation periods according to first-line EGFR-TKI.

Characteristic	Erlotinib (n=71)	Afatinib (n=36)	Osimertinib (n=14)	Dacomitinib (n=3)
Age, median (range), years	68 (43–86)	64.5 (35–85)	63 (30–78)	64 (59–67)
Female sex, n (%)	45 (63.4)	24 (66.7)	9 (64.3)	1 (33.3)
Smoking history, n (%)	21 (29.6)	9 (25.0)	2 (14.3)	1 (33.3)
ECOG PS 0, n (%)	2 (2.8)	2 (5.6)	0 (0.0)	0 (0.0)
ECOG PS 1, n (%)	50 (70.4)	27 (75.0)	8 (57.1)	3 (100.0)
ECOG PS 2, n (%)	19 (26.8)	7 (19.4)	6 (42.9)	0 (0.0)
Adenocarcinoma, n (%)	67 (94.4)	35 (97.2)	14 (100.0)	2 (66.7)
Others histopathology, n (%)	4 (5.6)	1 (2.8)	0 (0.0)	1 (33.3)
EGFR exon 19 deletion, n (%)	56 (78.9)	17 (47.2)	8 (57.1)	0 (0.0)
EGFR exon 21 L858R, n (%)	14 (19.7)	13 (36.1)	6 (42.9)	2 (66.7)
Uncommon EGFR mutation, n (%)	0 (0.0)	7 (19.4)	0 (0.0)	1 (33.3)
Metastatic disease at initial diagnosis, n (%)	53 (74.6)	25 (69.4)	9 (64.3)	1 (33.3)
First-line EGFR-TKI initiation years	2017–2024	2019–2024	2024–2025	2022–2024
Initiated in 2017–2020, n (%)	20 (28.2)	13 (36.1)	0 (0.0)	0 (0.0)
Initiated in 2021–2023, n (%)	40 (56.3)	21 (58.3)	0 (0.0)	1 (33.3)
Initiated in 2024–2025, n (%)	11 (15.5)	2 (5.6)	14 (100.0)	2 (66.7)

Values are presented as n (%) unless otherwise specified. "Others" includes squamous cell carcinoma, adenosquamous carcinoma, and NSCLC–not otherwise specified (NOS). Uncommon EGFR mutations comprise S768I, G719X, and L861Q. Treatment initiation periods refer to the date of first-line EGFR-TKI initiation for metastatic disease. Abbreviations: ECOG PS, Eastern Cooperative Oncology Group performance status; EGFR-TKI, epidermal growth factor receptor tyrosine kinase inhibitor.

Supplementary Table S2. Multivariable Cox regression analyses of overall survival and progression-free survival using clinically selected covariates.

Variable	OS adjusted HR (95% CI)	p-value	PFS adjusted HR (95% CI)	p-value
Sex	1.180 (0.549–2.537)	0.672	1.152 (0.615–2.159)	0.659
Smoking status	1.288 (0.619–2.683)	0.498	0.894 (0.486–1.646)	0.720
ECOG performance status	4.592 (2.563–8.227)	**<0.001**	1.636 (1.005–2.663)	**0.048**
Histopathological group	5.614 (2.066–15.254)	**0.001**	2.957 (1.164–7.509)	**0.023**
EGFR mutation subtype (overall)	—	**0.015**	—	0.309
Exon 19 deletion	Reference	—	Reference	—
L858R	1.433 (0.830–2.475)	0.197	1.095 (0.689–1.738)	0.701
Uncommon mutations	3.477 (1.447–8.354)	**0.005**	1.888 (0.831–4.288)	0.129
Brain metastasis	0.968 (0.506–1.850)	0.922	0.848 (0.499–1.443)	0.544
Liver metastasis	1.122 (0.535–2.355)	0.761	1.146 (0.614–2.140)	0.668
Bone metastasis	2.008 (1.146–3.518)	**0.015**	1.344 (0.853–2.119)	0.202
Pleural metastasis	0.773 (0.458–1.304)	0.335	1.576 (1.012–2.455)	**0.044**
Metastatic disease at diagnosis	1.431 (0.846–2.419)	0.182	0.945 (0.587–1.524)	0.818

Abbreviations: OS, overall survival; PFS, progression-free survival; HR, hazard ratio; CI, confidence interval; ECOG, Eastern Cooperative Oncology Group; EGFR, epidermal growth factor receptor.

All covariates were entered simultaneously into supplementary Cox models irrespective of their univariable p-values. The overall EGFR mutation subtype p-value is from the 2-df Wald test; exon 19 deletion is the reference category for EGFR comparisons.
For sex, smoking status, ECOG performance status, histopathological group, metastasis indicators, and metastatic disease at diagnosis, HR direction follows the original SPSS coding. Reference categories should be confirmed from the SPSS variable coding before journal submission.
The histopathological “Others” category comprises 3 NSCLC-NOS, 2 squamous cell carcinomas, and 1 adenosquamous carcinoma (n=6). Findings for this small heterogeneous group and the uncommon EGFR subgroup (n=8) are exploratory.
